# Supplementary material for: Trends in smoking initiation and cessation over a century in two Australian cohorts
Source: PLoS One. 2024 Sep 19;19(9):e0307386. doi: 10.1371/journal.pone.0307386 (PMC11412490; doi:10.1371/journal.pone.0307386)
Supplement: S1 Fig — (DOC) [file pone.0307386.s001.doc]

**S1 Fig**. Number of BHS and TAHS participants included in the analysis according to the period of data collection.
