# Supplementary material for: Trends in smoking initiation and cessation over a century in two Australian cohorts
Source: PLoS One. 2024 Sep 19;19(9):e0307386. doi: 10.1371/journal.pone.0307386 (PMC11412490; doi:10.1371/journal.pone.0307386)
Supplement: S1 Appendix — (DOC) [file pone.0307386.s002.doc]

**S1 Appendix.** Sensitivity analysis on sustained smoking cessation.

As the risk of relapse is high in the first year after quitting while it drastically drops afterwards (1), we performed a sensitivity analysis defining “sustained smoking cessation” as having quitted smoking for at least two years, with person-years re-calculated accordingly, in order to exclude subjects who might have quit smoking just for a brief time before the survey.

The analysis showed consistent age and time trends in smoking cessation compared to the main analysis, although the magnitude of the rates varied slightly (**Tables A and B**). Adjusted rates of sustained smoking cessation are reported in **Figure A**.

**Table A.** Crude rates of sustained smoking cessation and person-years at risk for males and females, by age group (1930–2005), calculated using the pooled dataset (TAHS + BHS).

|  | Males | | Females | |
| --- | --- | --- | --- | --- |
| Age group  (years) | Rate  (per 1,000/year) | Person-years | Rate  (per 1,000/year) | Person-years |
| 36-37 | 21.0 | 9,982 | 17.7 | 7,960 |
| 38-39 | 26.4 | 9,488 | 23.2 | 7,673 |
| 40-41 | 41.2 | 8,364 | 40.7 | 6,736 |
| 42-43 | 30.6 | 6,184 | 24.6 | 4,843 |
| 44-45 | 39.2 | 4,952 | 29.7 | 3,768 |
| 46-47 | 25.8 | 4,078 | 28.0 | 3,102 |
| 48-50 | 46.5 | 4,478 | 46.5 | 3,227 |

**Table B:** Crude rates of sustained smoking cessation per 1000/year (and person-years at risk), by sex, cohort and period. Cells with less than 100 person-years at risk are omitted.

|  | Males aged  Age 36-50 | | | Females  Age 36-50 | | |
| --- | --- | --- | --- | --- | --- | --- |
|  | BHS | TAHS | **Pooled** | BHS | TAHS | **Pooled** |
| 1930-1939 | 5.6  (1,241) |  | **5.6**  **(1,241)** | 4.3  (233) |  | **4.3**  **(233)** |
| 1940-1949 | 9.8  (2,662) |  | **9.8**  **(2,662)** | 10.1  (791) |  | **10.1**  **(791)** |
| 1950-1959 | 21.4  (4,021) |  | **21.4**  **(4,021)** | 10.6  (1,972) |  | **10.6**  **(1,972)** |
| 1960-1969 | 26.9  (4,237) |  | **26.9**  **(4,237)** | 13.0  (2,774) |  | **13.0**  **(2,774)** |
| 1970-1979 | 37.6  (2,316) |  | **37.6**  **(2,316)** | 21.4  (1,727) |  | **21.4**  **(1,727)** |
| 1980-1989 | 61.7  (1,118) | 40.7  (885) | **52.4**  **(2,003)** | 37.6  (797) | 35.1  (768) | **36.4**  **(1,565)** |
| 1990-1999 | 44.4  (3,061) | 28.4  (12,448) | **31.5**  **(15,509)** | 44.1  (2,970) | 23.5  (10,851) | **27.9**  **(13,821)** |
| 2000-2005 | 49.6  (1,732) | 34.7  (12,430) | **36.5**  **(14,162)** | 54.7  (1,810) | 32.3  (11,267) | **35.4**  **(13,077)** |

**Figure A.** Estimated trends in sustained smoking cessation with 95% confidence intervals, by sex.*

* blue lines: males; red lines: females. Generalized linear models with negative binomial distribution, logarithmic link function, an offset for log person-years, and adjusted for study group, age, and age^2^; time was modelled using natural splines with 3 equally spaced inner knots. The bump in the curves in 1975-1984 was due to having no person-years at risk for 1980 and 1981, and few cases of sustained quitting in general during the period

**References**

1) Garcia-Rodriguez O, Secades-Villa R, Florez-Salamanca L, Okuda M, Liu SM, Blanco C. Probability and predictors of relapse to smoking: results of the National Epidemiologic Survey on Alcohol and Related Conditions (NESARC). Drug Alcohol Depend 2013; 132:479–85. <https://doi.org/10.1016/j.drugalcdep.2013.03.008> PMID: 23570817
