# Supplementary material for: Trends in smoking initiation and cessation over a century in two Australian cohorts
Source: PLoS One. 2024 Sep 19;19(9):e0307386. doi: 10.1371/journal.pone.0307386 (PMC11412490; doi:10.1371/journal.pone.0307386)
Supplement: S1 Table — (DOC) [file pone.0307386.s005.doc]

**S1 Table.** Distribution of the characteristics of BHS participants by study wave.

|  | BHS waves (years) | | | | | | | |
| --- | --- | --- | --- | --- | --- | --- | --- | --- |
| Characteristics | 1966 | 1969 | 1972 | 1975 | 1978 | 1981 | 1987 | 2010–2015 |
| Number of participants | 3394 | 3672 | 3879 | 3611 | 3931 | 3925 | 1057 | 5080 |
| Participation  rate (%) | 91% | 90% | 86% | 76% | 74% | 64% | 54% | 62% |
| Women (n, %) | 1723 (50.8) | 1906 (51.8) | 2032 (52.3) | 1947 (53.6) | 2177 (54.3) | 2142 (54.4) | 601 (53.8) | 2800 (54.8) |
| Birth year  (median, min–max) | 1920  (1873–1946) | 1921  (1875–1950) | 1925  (1879–1955) | 1927  (1881–1959) | 1929  (1881–1960) | 1930  (1884–1965) | 1915  (1889–1947) | 1954  (1944–1964) |
| Age, years  (median, min–max) | 46 (20–93) | 48 (19–94) | 47 (17–93) | 48 (16–94) | 49 (18–97) | 51 (16–97) | 73 (40–98) | 56 (46–66) |
| Ever smoking (n, %) | 1801 (53.1) | 1929 (52.5) | 1871 (48.2) | 1568 (43.4) | 1938 (49.3) | 1852 (47.2) | 533 (50.4) | 2696 (53.1) |
| Age at initiation,  years (mean±SD) | 19.7±7.3 | 19.8±7.1 | 19.2±6.4 | 18.7±5.6 | 18.7±5.9 | 18.7±5.9 | 19.5±7.3 | 17.1±3.9 |
| Former smoking (n, %) | 516 (28.7) | 637 (33.0) | 671 (35.9) | 630 (40.2) | 991 (51.4)* | 1084 (58.7)* | 415 (77.9) | 2178 (81.1)* |
| Age at cessation,  years (mean±SD) | 40.7±15.1 | 41.1±14.7 | 41.3±15.1 | 41.5±14.9 | 41.7±15.6 | 41.4±14.9 | 52.6±15.0 | 36.5±12.1 |

* at this time point information on the status of former smoking was missing for some subjects (n<10)
