# Supplementary material for: Trends in smoking initiation and cessation over a century in two Australian cohorts
Source: PLoS One. 2024 Sep 19;19(9):e0307386. doi: 10.1371/journal.pone.0307386 (PMC11412490; doi:10.1371/journal.pone.0307386)
Supplement: S2 Table — (DOC) [file pone.0307386.s006.doc]

**S2 Table.** Values of the Bayesian Information Criterion (BIC) for the different numbers of knots tested in the natural spline functions for period, by analysis and sex.

| **Analysis** | **Age group** | **N. of knots** | **BIC values for males** | **BIC values for females** |
| --- | --- | --- | --- | --- |
| Smoking initiation | 11-15 | 3 | 1771.26 | 1074.86 |
|  |  | 4 | 1766.38* | 1019.79 |
|  |  | 5 | 1771.91 | 1008.09* |
|  | 16-20 | 3 | 2299.95* | 2136.18* |
|  |  | 4 | 2300.24 | 2140.84 |
|  |  | 5 | 2305.79 | 2146.94 |
|  | 21-35 | 3 | 1345.23 | 1549.24* |
|  |  | 4 | 1344.38* | 1553.75 |
|  |  | 5 | 1347.04 | 1560.51 |
| Smoking cessation  (main analysis) | 36-50 | 3 | 2922.06* | 2205.60* |
|  |  | 4 | 2924.55 | 2209.95 |
|  |  | 5 | 2932.40 | 2215.96 |
| Smoking cessation  (sensitivity analysis) | 36-50 | 3 | 2824.25* | 2148.05* |
|  |  | 4 | 2825.84 | 2151.11 |
|  |  | 5 | 2833.71 | 2157.48 |

* selected number of knots from the model with the lowest BIC value
