# Supplementary material for: Trends in smoking initiation and cessation over a century in two Australian cohorts
Source: PLoS One. 2024 Sep 19;19(9):e0307386. doi: 10.1371/journal.pone.0307386 (PMC11412490; doi:10.1371/journal.pone.0307386)
Supplement: S3 Table — a analysis restricted to the subjects who reported to be ever smokers at both waves under comparison. (DOC) [file pone.0307386.s007.doc]

**S3 Table.** Comparison of age at smoking initiation reported at different BHS waves. ^a^

|  | 1^st^ wave | 2^nd^ wave | N of people smoking | Later initiation at 2^nd^ wave, n (%) | Same age at initiation (±1 year), n (%) | Earlier initiation at 2^nd^ wave, n (%) | Age at initiation at 1^st^ wave (years), mean±SD | Age at initiation at 2^nd^ wave (years), mean±SD | Spearman’s rank correlation coefficient |
| --- | --- | --- | --- | --- | --- | --- | --- | --- | --- |
| Waves using different items | 1969 | 1972 | 1289 | 209 (16.2) | 856 (66.4) | 224 (17.4) | 19.2±5.8 | 19.2±6.0 | 0.82 |
|  | 1975 | 1978 | 916 | 113 (12.3) | 636 (69.3) | 167 (18.2) | 18.9±5.5 | 18.7±5.7 | 0.84 |
|  | 1981 | 1990 | 119 | 25 (21.1) | 82 (68.9) | 12 (10.1) | 17.0±2.8 | 17.5±2.9 | 0.73 |
|  | 1987 | 2005 | 22 | 7 (31.8) | 15 (68.2) | 0 (0.0) | 21.3±7.3 | 22.2±6.9 | 0.95 |
|  | 2005 | 2010 | 265 | 25 (9.4) | 195 (73.6) | 45 (17.0) | 17.3±3.2 | 17.0±3.2 | 0.73 |
| Waves using similar items | 1966 | 1969 | 1364 | 278 (20.4) | 859 (62.9) | 227 (16.6) | 19.4±6.7 | 19.6±6.5 | 0.82 |
|  | 1972 | 1975 | 1010 | 113 (11.2) | 788 (78.0) | 109 (10.8) | 18.9±5.4 | 19.0±5.6 | 0.89 |
|  | 1978 | 1981 | 1124 | 142 (12.6) | 831 (73.9) | 151 (13.4) | 18.8±5.7 | 18.8±6.1 | 0.87 |
|  | 1981 | 1987 | 295 | 56 (18.9) | 182 (61.7) | 57 (19.3) | 19.7±7.1 | 19.5±7.1 | 0.87 |
|  | 1978 | 1987 | 267 | 49 (18.4) | 160 (59.9) | 58 (21.7) | 19.4±6.1 | 19.4±7.2 | 0.85 |

^a^ analysis restricted to the subjects who reported to have ever smoked at both waves under comparison
