# Supplementary material for: Trends in smoking initiation and cessation over a century in two Australian cohorts
Source: PLoS One. 2024 Sep 19;19(9):e0307386. doi: 10.1371/journal.pone.0307386 (PMC11412490; doi:10.1371/journal.pone.0307386)
Supplement: S4 Table — a analysis restricted to the subjects who reported to be quitters at both waves under comparison. (DOC) [file pone.0307386.s008.doc]

**S4 Table. Comparison of age at smoking cessation reported at different BHS waves. ^a^**

|  | 1^st^ wave | 2^nd^ wave | N of people smoking | Later cessation at 2^nd^ wave, n (%) | Same age at cessation (±1 year), n (%) | Earlier cessation at 2^nd^ wave, n (%) | Age at cessation at 1^st^ wave (years), mean±SD | Age at cessation at 2^nd^ wave (years), mean±SD | Spearman’s rank correlation coefficient |
| --- | --- | --- | --- | --- | --- | --- | --- | --- | --- |
| Waves using different items | 1969 | 1972 | 348 | 64 (18.4) | 217 (62.4) | 67 (19.2) | 40.3±13.7 | 40.3±13.2 | 0.95 |
|  | 1975 | 1978 | 317 | 87 (27.4) | 186 (58.7) | 44 (13.9) | 42.4±14.0 | 43.5±14.2 | 0.95 |
|  | 1981 | 2010 | 40 | 12 (30.0) | 22 (55.0) | 6 (15.0) | 24.6±4.7 | 27.5±9.1 | 0.60 |
| Waves using similar items | 1966 | 1969 | 293 | 68 (23.2) | 156 (53.2) | 69 (23.6) | 40.5±13.9 | 40.3±13.4 | 0.93 |
|  | 1972 | 1975 | 303 | 47 (15.5) | 218 (71.9) | 38 (12.5) | 41.5±14.0 | 41.7±14.1 | 0.96 |
|  | 1978 | 1981 | 543 | 123 (22.7) | 322 (56.3) | 98 (18.0) | 42.3±14.5 | 42.5±14.4 | 0.96 |
|  | 1981 | 1987 | 178 | 48 (26.9) | 90 (50.6) | 40 (22.5) | 49.2±12.8 | 49.1±13.9 | 0.89 |
|  | 1978 | 1987 | 151 | 52 (34.4) | 64 (42.4) | 35 (23.2) | 48.8±12.8 | 49.1±13.8 | 0.89 |

^a^ analysis restricted to the subjects who reported to have quitted smoking at both waves under comparison
