# Supplementary material for: Trends in smoking initiation and cessation over a century in two Australian cohorts
Source: PLoS One. 2024 Sep 19;19(9):e0307386. doi: 10.1371/journal.pone.0307386 (PMC11412490; doi:10.1371/journal.pone.0307386)
Supplement: S5 Table — a cells with less than 100 person-years at risk were omitted. (DOC) [file pone.0307386.s009.doc]

**S5 Table.** Crude rates of smoking initiation per 1000/year and person-years at risk for males by age group, cohort and period. ^a^

|  | Age 11–15 | | | Age 16–20 | | | Age 21–35 | | |
| --- | --- | --- | --- | --- | --- | --- | --- | --- | --- |
|  | BHS | TAHS | **Pooled** | BHS | TAHS | **Pooled** | BHS | TAHS | **Pooled** |
| 1910–1919 | 42.2  (2,014) |  | **42.2**  **(2,014)** | 148.8  (773) |  | **148.8**  **(7,73)** | 61.9  (388) |  | **61.9**  **(388)** |
| 1920–1929 | 39.4  (3,199) |  | **39.4**  **(3,199)** | 131.9  (1,788) |  | **131.9**  **(1,788)** | 60.3  (1,061) |  | **60.3**  **(1,061)** |
| 1930–1939 | 28.4  (3,315) |  | **28.4**  **(3,315)** | 135.3  (2,314) |  | **135.3**  **(2,314)** | 41.7  (2,376) |  | **41.7**  **(2,376)** |
| 1940–1949 | 37.6  (2,737) |  | **37.5**  **(2,744)** | 173.9  (1,788) |  | **173.9**  **(1,788)** | 45.9  (2,674) |  | **45.9**  **(2,674)** |
| 1950–1959 | 30.6  (4,082) | 38.9  (874) | **32.1**  **(4,956)** | 124.2  (1,917) |  | **124.5**  **(1,992)** | 16.5  (2,538) |  | **16.5**  **(2,542)** |
| 1960–1969 | 37.7  (7,898) | 40.2  (8,903) | **39.0**  **(16,801)** | 120.3  (4,330) | 127.6  (2,139) | **122.7**  **(6,469)** | 16.1  (3,729) | 26.8  (411) | **17.1**  **(4,140)** |
| 1970–1979 | 57.6  (4,653) | 58.2  (30,383) | **58.1**  **(35,036)** | 105.3  (4,152) | 109.1  (15,203) | **108.2**  **(19,355)** | 6.8  (7,108) | 15.4  (5,654) | **10.6**  **(12,762)** |
| 1980–1989 |  | 91.2  (1,370) | **91.2**  **(1,370)** | 70.3  (1,038) | 78.7  (8,222) | **77.8**  **(9,260)** | 5.2  (7,130) | 9.4  (28,151) | **8.5**  **(35,281)** |
| 1990–1999 |  |  |  |  |  |  | 1.9  (2,613) | 2.5  (20,279) | **2.4**  **(22,892)** |

^a^ cells with less than 100 person-years at risk were omitted
