# Supplementary material for: Trends in smoking initiation and cessation over a century in two Australian cohorts
Source: PLoS One. 2024 Sep 19;19(9):e0307386. doi: 10.1371/journal.pone.0307386 (PMC11412490; doi:10.1371/journal.pone.0307386)
Supplement: S6 Table — a cells with less than 100 person-years at risk were omitted. (DOC) [file pone.0307386.s010.doc]

**S6 Table.** Crude rates of smoking initiation per 1000/year (and person-years at risk) for females by age group, cohort and period. ^a^

|  | Age 11–15 | | | Age 16–20 | | | Age 21–35 | | |
| --- | --- | --- | --- | --- | --- | --- | --- | --- | --- |
|  | BHS | TAHS | **Pooled** | BHS | TAHS | **Pooled** | BHS | TAHS | **Pooled** |
| 1910–1919 | 0.5  (1,993) |  | **0.5**  **(1,993)** | 12.6  (1,266) |  | **12.6**  **(1,266)** | 3.4  (1,479) |  | **3.4**  **(1,479)** |
| 1920–1929 | 3.2  (3,419) |  | **3.2**  **(3,419)** | 17.2  (2,677) |  | **17.2**  **(2,677)** | 6.6  (3,783) |  | **6.6**  **(3,783)** |
| 1930–1939 | 5.3  (3,784) |  | **5.3**  **(3,784)** | 42.8  (3,548) |  | **42.8**  **(3,548)** | 13.4  (6,713) |  | **13.4**  **(6,713)** |
| 1940–1949 | 6.9  (3,162) |  | **6.9**  **(3,169)** | 63.8  (3,009) |  | **63.8**  **(3,009)** | 16.9  (7,527) |  | **16.9**  **(7,527)** |
| 1950–1959 | 7.3  (4,382) | 8.5  (706) | **7.5**  **(5,088)** | 62.7  (2,726) |  | **62.3**  **(2,824)** | 13.3  (6,549) |  | **13.3**  **(6,556)** |
| 1960–1969 | 17.6  (9,799) | 17.8  (8,431) | **17.7**  **(18,230)** | 81.4  (6,239) | 87.6  (2,317) | **83.1**  **(8,556)** | 10.2  (6,649) | 31.3  (479) | **11.6**  **(7,128)** |
| 1970–1979 | 47.4  (6,011) | 51.1  (29,404) | **50.5**  **(35,415)** | 97.5  (6,039) | 111.9  (15,555) | **107.9**  **(21,594)** | 7.3  (11,327) | 13.6  (6,540) | **9.6**  **(17,867)** |
| 1980–1989 |  | 87.8  (1,287) | **87.8**  **(1,287)** | 70.9  (1,212) | 81.6  (7,723) | **80.1**  **(8,935)** | 4.9  (10,471) | 8.2  (28,606) | **7.3**  **(39,077)** |
| 1990–1999 |  |  |  |  |  |  | 1.8  (3,394) | 2.2  (19,406) | **2.1**  **(22,800)** |

^a^ cells with less than 100 person-years at risk were omitted
