# Supplementary material for: Trends in smoking initiation and cessation over a century in two Australian cohorts
Source: PLoS One. 2024 Sep 19;19(9):e0307386. doi: 10.1371/journal.pone.0307386 (PMC11412490; doi:10.1371/journal.pone.0307386)
Supplement: S7 Table — (DOC) [file pone.0307386.s011.doc]

**S7 Table.** Crude rates of smoking cessation per 1000/year (and person-years at risk) for males, by cohort and period.

|  | Age 16–35 | | | Age 36–50 | | | Age 51–65 | | |
| --- | --- | --- | --- | --- | --- | --- | --- | --- | --- |
|  | BHS | TAHS | **Pooled** | BHS | TAHS | **Pooled** | BHS | TAHS | **Pooled** |
| 1930–1939 | 1.7  (8,962) |  | **1.7**  **(8,962)** | 3.8  (2,076) |  | **3.8**  **(2,076)** |  |  |  |
| 1940–1949 | 4.4  (9,861) |  | **4.4**  **(9,861)** | 6.1  (4,750) |  | **6.1**  **(4,750)** | 10.5  (1,044) |  | **10.5**  **(1,044)** |
| 1950–1959 | 6.9  (8,265) |  | **6.9**  **(8,265)** | 13.0  (6,689) |  | **13.0**  **(6,689)** | 15.8  (2,717) |  | **15.8**  **(2,717)** |
| 1960–1969 | 11.8  (9,538) | 5.2  (2,668) | **10.4**  **(12,206)** | 19.9  (5,968) |  | **19.9**  **(5,968)** | 21.8  (4,580) |  | **21.8**  **(4,580)** |
| 1970–1979 | 25.5  (12,129) | 12.9  (21,990) | **17.4**  **(34,111)** | 34.8  (2,866) |  | **34.8**  **(2,866)** | 40.1  (3,242) |  | **40.1**  **(3,242)** |
| 1980–1989 | 32.3  (7,902) | 20.2  (42,439) | **22.1**  **(50,322)** | 44.8  (1,872) | 30.7  (1,170) | **39.4**  **(3,042)** | 54.1  (499) |  | **54.1**  **(499)** |
| 1990–1999 | 33.4  (2,155) | 26.8  (21,907) | **27.4**  **(24,050)** | 32.8  (4,350) | 26.6 (13,667) | **28.1**  **(18,017)** | 20.3  (148) | 39.4  (279) | **32.8**  **(427)** |
| 2000–2005 |  | 22.7  (660) | **22.8**  **(659)** | 40.2  (2,160) | 33.4 (14,795) | **34.3 (16,955)** | 49.2  (2,542) | 36.6  (2,077) | **43.6**  **(4,612)** |
