# Supplementary material for: Trends in smoking initiation and cessation over a century in two Australian cohorts
Source: PLoS One. 2024 Sep 19;19(9):e0307386. doi: 10.1371/journal.pone.0307386 (PMC11412490; doi:10.1371/journal.pone.0307386)
Supplement: S8 Table — (DOC) [file pone.0307386.s012.doc]

**S8 Table.** Crude rates of smoking cessation per 1000/year (and person-years at risk) for females, by cohort and period.

|  | Age 16–35 | | | Age 36–50 | | | Age 51–65 | | |
| --- | --- | --- | --- | --- | --- | --- | --- | --- | --- |
|  | BHS | TAHS | **Pooled** | BHS | TAHS | **Pooled** | BHS | TAHS | **Pooled** |
| 1930–1939 | 1.3  (3,956) |  | **1.3**  **(3,956)** | 2.5  (403) |  | **2.4**  **(403)** |  |  |  |
| 1940–1949 | 4.2  (5,776) |  | **4.2**  **(5,776)** | 5.8  (1,357) |  | **5.8**  **(1,357)** | 5.0  (201) |  | **5.0**  **(201)** |
| 1950–1959 | 6.5  (5,358) |  | **6.5**  **(5,358)** | 6.6  (3,162) |  | **6.6**  **(3,162)** | 5.6  (712) |  | **5.6**  **(712)** |
| 1960–1969 | 13.6  (6,984) | 5.8  (1,898) | **12.0**  **(8,869)** | 9.9  (3,904) |  | **9.9**  **(3,904)** | 19.0  (1,952) |  | **19.0**  **(1,952)** |
| 1970–1979 | 26.8  (11,464) | 11.3  (18,583) | **17.2**  **(30,021)** | 19.3  (2,121) |  | **19.3**  **(2,121)** | 32.5  (2,369) |  | **32.5**  **(2,369)** |
| 1980–1989 | 39.6  (8,612) | 22.2  (38,590) | **25.4**  **(47,185)** | 23.6  (1,438) | 28.7  (939) | **25.6**  **(2,377)** | 44.9  (490) |  | **44.9**  **(490)** |
| 1990–1999 | 40.5  (2,296) | 26.8  (19,936) | **28.2**  **(22,229)** | 31.18 (4,201) | 22.2 (11,561) | **24.6 (15,762)** | 29.6  (135) | 32.1  (218) | **31.7**  **(347)** |
| 2000–2005 |  | 53.2  (526) | **53.2**  **(526)** | 44.7  (2,237) | 32.5 (13,254) | **34.2 (15,491)** | 57.0  (2,299) | 29.6  (1,793) | **45.0**  **(4,070)** |
