# Supplementary material for: Trends in smoking initiation and cessation over a century in two Australian cohorts
Source: PLoS One. 2024 Sep 19;19(9):e0307386. doi: 10.1371/journal.pone.0307386 (PMC11412490; doi:10.1371/journal.pone.0307386)
Supplement: Attachment — Submitted filename: Responses to comments (R1).docx [file pone.0307386.s013.docx]

**PONE-D-24-00230**

**Trends in smoking initiation and cessation over a century in two Australian cohorts**

Responses to comments

# **Editor**

C1) After careful consideration, we feel that it has merit but does not fully meet PLOS ONE’s publication criteria as it currently stands. Therefore, we invite you to submit a revised version of the manuscript that addresses the points raised during the review process.

In particular, you should address the methodological comments by our reviewers, the question of representativeness and generalizability of results, potential biases associated with restriction to ages 35 - 50 years, and the possible effects and timing of tobacco-control measures in Australia.

We express our sincere gratitude to the Editor and the reviewers for their valuable comments on our manuscript. We have carefully considered the criticisms and suggestions provided and have implemented revisions aimed at enhancing the quality and clarity of the manuscript. Throughout this process, we have documented the changes made and have responded to each comment in the letter below. Here are the main changes we have implemented:

1) We have conducted a sensitivity analysis using a new definition of smoking cessation, requiring subjects to have quit smoking for at least 2 years. The results of these analyses are fully documented in the supplement (**S1 Appendix**). Although there were slight changes in the magnitude (absolute values) of smoking cessation rates, the age and temporal trends remained similar to those observed in the main analysis.
2) We have provided the crude rates of smoking cessation for age ranges that were not previously considered i.e., 16-35 years and 51-65 years (**S7 and S8 Tables**); moreover, we have better explained the reasons why the adjusted analyses for smoking cessation were not conducted for these age groups, and discussed the implications for study validity of having focused the analyses on subjects aged 35-50 years.
3) We have expanded the description of the timing of tobacco-control measures in Australia and emphasized that the current study is descriptive in nature, which implies that any inference regarding the relationship between policy and changes in smoking initiation/cessation patterns should be made with caution.

# **Journal requirements**

When submitting your revision, we need you to address these additional requirements.

1. Please ensure that your manuscript meets PLOS ONE's style requirements, including those for file naming. The PLOS ONE style templates can be found at

https://journals.plos.org/plosone/s/file?id=wjVg/PLOSOne_formatting_sample_main_body.pdf and

https://journals.plos.org/plosone/s/file?id=ba62/PLOSOne_formatting_sample_title_authors_affiliations.pdf

We have checked that the manuscript met PLOS ONE’s style requirements, including those for file naming.

2. Thank you for stating the following financial disclosure:

“Article processing charges were supported by the special fund at the University of Verona dedicated to Open Access publications”

Please state what role the funders took in the study. If the funders had no role, please state: "The funders had no role in study design, data collection and analysis, decision to publish, or preparation of the manuscript."

If this statement is not correct you must amend it as needed.

Please include this amended Role of Funder statement in your cover letter; we will change the online submission form on your behalf.

We have reported the new funding statement in the cover letter as follows:

*Article processing charges were supported by the special fund at the University of Verona dedicated to Open Access publications. The funder had no role in study design, data collection and analysis, decision to publish, or preparation of the manuscript.*

3. Thank you for stating the following in the Acknowledgments Section of your manuscript:

“Article processing charges were supported by the special fund at the University of Verona dedicated to Open Access publications.”

We note that you have provided funding information that is currently declared in your Funding Statement. However, funding information should not appear in the Acknowledgments section or other areas of your manuscript. We will only publish funding information present in the Funding Statement section of the online submission form.

Please remove any funding-related text from the manuscript and let us know how you would like to update your Funding Statement. Currently, your Funding Statement reads as follows:

“Article processing charges were supported by the special fund at the University of Verona dedicated to Open Access publications”

Please include your amended statements within your cover letter; we will change the online submission form on your behalf.

The acknowledgement section has been deleted from the manuscript.

# **Reviewer #1**

The authors describe smoking initiation and cessation among two Australian cohorts (Tasmanian Longitudinal Health Study (TAHS) and Busselton Health Study (BHS)) who were 7 years old in 1966 and 1968 and followed for 40-50 years.

Thanks for the time and attention you dedicated in reviewing the manuscript. We appreciated your insightful comments and acknowledged your criticisms. We have revised the manuscript according to your suggestions, as well as those by the other reviewers.

In particular, we have added more data to show the temporal trends of smoking cessation at ages that were not considered in the previous analysis. Please find a point-by-point reply below.

**C1.1)** The most recent data were collected in 2005, nearly 20 years ago and well before the e-cigarette era, which radically changed youth nicotine addiction patterns. The authors briefly note this in the abstract and in the main text at line 391. The development of e-cigarettes, however, needs to be more carefully taken into account when interpreting the relevance of the results to public health policy making today. This does not invalidate the findings as far as they go, but require more cautious conclusions about current relevance of those findings.

We acknowledge that one limitation of our study is the use of non-recent data. While we believe that our manuscript offers valuable insights into historical trends in smoking habits over an extended period (9 decades for smoking initiation and 8 decades for smoking cessation in the crude analysis), we recognize that these data do not capture the most recent period, particularly after the introduction of new nicotine-containing products. In the revised version of the manuscript, we have placed additional emphasis on this limitation (lines 439-444 in the tracked-changes version).

**C1.2)** The results were pooled into a single analysis. There is nothing wrong in principle with doing this, but the smoking questions were slightly different in the two studies. How they were combined and any associated limitations and uncertainties need to be described in more detail.

In the revised manuscript, we have provided clearer explanations about data pooling (lines 152-158) and further discussed the limitations and uncertainties of our approach (lines 439-444).

**C1.3)** The authors censored data for people who stopped smoking before age 35. This is a major problem, since many adolescents who experiment with cigarettes stop by young adulthood. To provide an accurate balance between initiation and cessation, the full range of cessation ages need to be included.

In the revised manuscript, we have added the crude cessation rates for age categories that were not covered in the original submission (ages 16-35 and 51-65 years). Please find these results in **S7 Table** for men, and **S8 Table** for women.

Of note, the crude cessation rates among male subjects aged 51-65 years showed a similar pattern compared to the 36-50 years-old group (**S7 Table**). In fact, we observed an increase in cessation from 1930-1939, a peak in 1980-1989, and a subsequent slight decline. A similar pattern was seen for the 16-35-year age group, except that the peak was reached later in 1990-1999.

Among women, the increasing trend was similar from 1930-1939 to 1970-1979; greater variability in the cessation rates was seen afterwards compared to males (**S8 Table**).

Part of the differences in cessation rates in the younger and older age groups, compared to the 35-50-year age group, is a consequence of having sparse data for 2000-2005 in the 16-50 age group (500-600 person-years over a 5-year period) and in 1980-1989 for the 51-65 age group (500 person-years over a 10-year period). Due to this issue of sparse data, we did not perform an adjusted analysis for smoking cessation for these age ranges, since the criterion of having at least 100 person-years per calendar year of the study period was not fulfilled.

In the revised manuscript, we have further acknowledged that having estimated adjusted rates of smoking cessation only for ages 36-60 years was a study limitation (see lines 399-404). This limitation arose from the secondary use of data that were not expressly collected for the purpose of investigating temporal trends in smoking habits, and therefore did not cover all periods and age groups in the same way. We encountered a similar issue with sparse data in our previous work on smoking cessation in Europe (Pesce et al., PLOS ONE 2019), where the same age range was eventually considered.

As a consequence of the restriction of the age range to 36-50 years, inference from our study can only address this specific age range and cannot be extrapolated to other age groups (lines 414-416). In other words, generalizing our findings to other age ranges would not be appropriate. However, it is important to note that this does not inherently cause selection bias, which is also evident from the comparison of crude rates for the three age groups in **S7 and S8 Tables**.

**C1.4)** How were the statistical adjustments made (line 186)?

We agree that the methodology needed further clarifications. We have improved the description of the statistical adjustment as suggested (lines 225-239). Moreover, we have specified the type of model, number of knots, and variables included in the adjusted analyses as a note to **Figures 2 and 3**.

**C1.5)** RE: Data availability: The authors state, "All relevant data are within the manuscript and its Supporting Information files," but I don't see the actual raw data. The raw data also needs to be made available per PLOS ONE policies.

Thanks for highlighting this inconsistency. The minimal data sets to replicate the analyses on smoking initiation and cessation are now enclosed in the Supplementary materials as **S1 and S2 Datasets**, respectively.

# **Reviewer #2**

Thank you for the possibility to peer-review the abovementioned study. The aim of the study was to investigate the trends of smoking initiation and cessation in Australia between 1910 and 2005. The topic is important as smoking remains one of the largest threats for public health. In this study, time trends, as well as gender and age effects were evaluated within nearly 30.000 participants of two population-based cohorts. The findings highlight that tobacco control policies have had less success among younger populations.

Thanks for the time and attention you dedicated in reviewing the manuscript. We appreciated your insightful comments and acknowledged your criticisms. We have revised the manuscript according to your suggestions, as well as those by the other reviewers.

In particular, we have strengthened the discussion on the generalisability of the study findings to the Australian population, and added more data to show the temporal trends of crude rates of smoking cessation at ages that were not considered in the previous analysis. Please find a point-by-point reply below.

**C2.1)** The authors mention in the introduction that, according to previous literature, public health campaigns to ban tobacco advertising, increasing the cost of cigarettes, health warnings on packaging and restriction of sales to minors have been successful in reducing the prevalence of smoking. Further, they conclude that the efficacy of tobacco control policies has not been uniform across all age groups, with a notable lack of success among the younger population since the 1970s. I was wondering what tobacco control policies have been made in Australia during the studied time period? Can you see the effects of certain policy changes? Have some policy changes been more effective than others? Can you say if some policy changes have better effects on the younger / older population than others?

*merged with later comment:*

What are the current tobacco policies in Australia? Can the authors make more explicit policy recommendations based on their findings?

Thanks for this valuable comment. In the revised manuscript, an addition has been made to paragraph 2 of the Introduction to outline the Australian measures and their effectiveness, adopted to counteract the smoking epidemic (lines 91-102 of the tracked-changes version):

*In Australia, public health groups began raising awareness of the detrimental health effects of smoking after the US Surgeon General’s report in 1967, however public health campaigns specifically targeting smoking such as the Quit Campaign only began in 1983, followed by health warnings on packaging and the National Tobacco Campaign in 1997 [9]. These measures have been associated with a steady reduction in the prevalence of smoking. In particular, health warnings, including pictorial representation of smoking related diseases on packaging and laws prohibiting the sale of cigarettes to minors, have all been associated with reduced smoking in teenagers [10]. These approaches and others, including increased cost of cigarettes, mass media controls and introduction of smoke-free environments, have been continued in Australia as part of the National Tobacco Strategies 2004-2012, 2012-2018 and 2023-2030 and state-based campaigns.*

As better explained in the revised manuscript (see lines 439-444), the present analysis consisted of a secondary use of data that were not expressly collected for the purpose of investigating temporal trends in smoking habits. The nature of the study was descriptive and, for this reason, we believe that inference regarding the relationship between policy implementation and changes in smoking initiation/cessation patterns was out of scope.

**C2.2)** The study was conducted in Australia. How do the authors consider the generalizability of the findings into other countries?

*merged with later comment:*

The authors state as the main finding of the study: “Between 1910 and 1999 up to 57% of people in Australia regularly smoked cigarettes.” How representative are these two cohorts of the total Australian population? Can the authors tell a bit more about the selection criteria for the Busselton Health Study cohort? Are children who attended school in Tasmania in 1968 similar to the total Australian population? There was some loss to follow up, does this affect the representativeness?

While the results of this study generally align with smoking trends observed in other parts of Australia and in high-income countries globally, it is important to note that due to its descriptive nature, this study was specific to the Australian context. Consequently, extrapolating the study findings to other populations may not be appropriate.

In the revised manuscript, we have further evaluated the matter of generalisability of the study findings to Australia, considering the local distribution of the study population, which was mainly concentrated in Western Australia and Tasmania (although 30% of the TAHS cohort now live across all the other states in Australia, see lines 137-138). Regional differences in smoking behaviour are evident for the Busselton Health Study, likely due to its presence and health data feed-back in the community for over 50 years. To further examine this study limitation, we have added some commentary in the Discussion – additional Paragraph 3 (lines 460-472):

*BHS and TAHS samples may not be fully representative of the broader Australian population, which could limit generalisability of our results. Regional centres have 3-5% higher prevalence of current smoking compared with major cities.[29] Compared with national Australian data,[27,28] the Busselton population showed higher proportions of adults formerly smoking in both genders between 1973 and 1984, and higher proportions of lifetime non-smoking individuals in women but not men.[26] Compared with a sample from the Australian National Health Survey 2011, Busselton adults aged 46-64 years studied between 2005 and 2007 were similar on a number of key health indicators, however the prevalence of current tobacco smoking in Busselton was 10%, lower than the national average for this age group at the time (15%).[29] Finally, the trends seen in smoking initiation and cessation may depend to some extent on changing demographics in the Busselton population over the last century.*

**C2.3)** Could the authors please add the (original) number of participants in the BHS cohort to the “study design and population” paragraph?

We have followed your suggestion (lines 149-151). The new sentence reads:

*The number of participants ranged from 1057 (1987) to 5080 (2010-2015) (S1 Table); participation rates were fairly high, ranging from 54% (1987) to 91% (1966).*

The reader can refer to **S1 Table** to get more details.

**C2.4)** The eligible age range for smoking cessation was 35-50 years to restrict to participants with a similar age distribution in the two cohorts. The age range for smoking initiation (11-35 years) was based on previous literature. Could the authors give information from previous literature, how many smokers do stop smoking at this chosen age? Does this age range cause selection bias to the results? How many smokers give up smoking, e.g., already in their late twenties or early thirties?

The authors recommend targeting young adult males to enhance smoking cessation in Australia. However, as discussed above, the study only investigated smoking cessation after the age of 35 years.

Thanks for our comment. In the revised manuscript, we have added the crude rates of smoking cessation for the age group 16-35 years in the BHS and TAHS studies, both considered separately and combined (**S7 and S8 Tables**). We were unfortunately unable to conduct an adjusted analysis covering this younger age group due to sparse data. In fact, the criterion of having at least 100 person-years per calendar year of the study period was not fulfilled (lines 230-234).

We appreciate the reviewer highlighting the issue of selection bias in identifying the suitable age range for analysis. It is important to note that the restriction of the age range to 36-50 years limits the generalisability of the study findings to other age groups, but it does not inherently cause selection bias. This is also evident from the comparison of crude rates across age groups (**S7 and S8 Tables)**.

In the revised manuscript, we have emphasized discussion of this issue (lines 399-404 and 414-416).

# **Reviewer #3**

Thank you for the opportunity to review the manuscript 'Trends in smoking initiation and cessation over a century in two Australian cohorts'.

The manuscript reports on the rates of initiation and cessation estimated from longitudinal cohort studies conducted in Australia beginning in the latter part of the 20th century. The authors estimated trends in the rate of initiation and cessation of regular smoking amongst those who never (regularly) smoked and those who do smoke, respectively.

The statistical analysis is not quite complete, with some additional work needed to satisfy a reasonable scientific standard for this type of analysis. I have a few criticisms/suggestions for the introduction and discussion as the literature review does not accurately reflect the available knowledge for the Australian context, and the strengths and limitations of the study could be amended. I'll provide feedback section-by-section. I believe the extent of the amendments would be best described as 'major revisions'.

Thanks for the time and attention you dedicated in reviewing the manuscript. We appreciated your insightful comments and acknowledged your criticisms. We have revised the manuscript according to your suggestions, as well as those by the other reviewers.

In particular, we have added a sensitivity analysis with an alternative definition of ‘sustained’ smoking cessation as recommended, added more data to show the temporal trends of crude rates of cessation at ages that were not considered in the original analysis, improved the Introduction and Discussion based on recent literature, and revised the text using inclusive language. Please find a point-by-point reply below.

\## Introduction

**C3.1)** 1st paragraph: The following reference is relevant to the 2nd sentences, providing an Australian estimate on the number of years of life lost for those who continue to smoke:

- Banks, E., Joshy, G., Weber, M.F. et al. Tobacco smoking and all-cause mortality in a large Australian cohort study: findings from a mature epidemic with current low smoking prevalence. BMC Med 13, 38 (2015). <https://doi.org/10.1186/s12916-015-0281-z>

In addition; it was also estimated (contemporaneously to reference [5] in the manuscript) that those in the UK who quit before age 40 largely avoid the loss:

- Pirie, K., Peto, R., Reeves, G. K., Green, J., & Beral, V. (2013). The 21^st^ century hazards of smoking and benefits of stopping: a prospective study of one million women in the UK. The Lancet, 381(9861), 133-141. <https://doi.org/10.1016/S0140-6736(12)61720-6>

In the revised manuscript, we have added the suggested references to Banks et al and Pirie et al.

**C3.2)** 2nd paragraph: References [2,7] are specific to the European setting, however this is not clear in the text. In fact, there are ongoing surveys which have estimates for adolescent smoking in Australia, the 'ASSAD' survey, see for example this report (either the 2017 or 2023 release should have been referenced): <https://www.health.gov.au/resources/publications/secondary-school-students-use-of-tobacco-and-e-cigarettes-2022-2023?language=en>

3rd paragraph: Another reference that is important to the (global) understanding of initiation and cessation rates is the study of Holford et al, in 2014:

- Holford, T. R., Levy, D. T., McKay, L. A., Clarke, L., Racine, B., Meza, R., ... & Feuer, E. J. (2014). Patterns of birth cohort–specific smoking histories, 1965-2009. American journal of preventive medicine, 46(2), e31-e37.

which has also been picked up in Canada as a basis for their own model (but not of particular interest for this paper).

The European context of references 2 and 7 has been noted in the revised manuscript and the ASSAD survey reference has been added to the revised manuscript. Moreover, the Holford et al. reference has been added.

**C3.3)** The authors highlight certain priority populations, however these have been revised as of 2021 in Australia (nor does reference [3] provided by the authors mention those in jail). Care should be taken that Aboriginal and Torres Strait Islander people is capitalised correctly - the Australian co-authors on this paper should be able to provide guidance on these sensitive practises.

The National Preventive Health Strategy 2021-2030 for Australia identifies the following priority populations:

- Aboriginal and Torres Strait Islander people

- Culturally and linguistically diverse (CALD)

- LGBTQI+

- people with mental illness

- people of low socioeconomic status

- people with disability

- rural, regional, and remote

Source: <https://www.health.gov.au/resources/publications/national-preventive-health-strategy-2021-2030?language=en>

I would also advise against the wording 'resistant to cessation'; this is suggestive of a white, straight, able-bodied 'default' that is superior in some way to the 'other'.

Instead I would rephrase the sentence on lines 94-97 to "Knowledge of such trends could help tailor public health interventions by identifying specific groups who will thus experience a disproportionate exposure to tobacco; such as Aboriginal and Torres Strait Islander people, culturally and racially marginalised, people with mental illness, and people living in rural, regional, and remote areas."

Thank you for pointing this out and for the suggested rephrasing of the text. We took your comment into account by removing the reference to people in jail and changing the sentence as suggested (lines 114-120 in the tracked-changes version).

**C3.4)** The ethics of _only_ discussing disparity and disadvantage is also questionable, i.e. progress should also be included:

- Lovett R, Thurber KA, Wright A, Maddox R, Banks E. Deadly progress: changes in Australian Aboriginal and Torres Strait Islander adult daily smoking, 2004-2015. Public Health Res Pract. 2017;27(5):e2751742. <https://doi.org/10.17061/phrp2751742>

This has been amended (lines 514-515).

**C3.5)** 4th paragraph: The Australia context is not as scarcely studied as the authors suggest. In addition to the references above (the study on mortality by Banks, the ASSAD survey), there is also a study on cessation rates by Gartner et al, from 2009, along with a newer pre-print that extends the work to cover the better part of 80 years:

- Gartner, C. E., Barendregt, J. J., & Hall, W. D. (2009). Predicting the future prevalence of cigarette smoking in Australia: how low can we go and by when?. Tobacco control, 18(3), 183-189.

- Wade, S., Weber, M. F., Sarich, P., Vaneckova, P., Behar-Harpaz, S., Ngo, P. J., ... & Caruana, M. (2022). Bayesian calibration of simulation models: A tutorial and an Australian smoking behaviour model. arXiv preprint arXiv:2202.02923.

And there is the following study on long-term trends in cross-sectional surveys of Australians (incl. the age at initiation, and the overall number that initiate):

- Vaneckova P, Wade S, Weber M, Murray JM, Grogan P, et al. (2021) Birth-cohort estimates of smoking initiation and prevalence in 20th century Australia: Synthesis of data from 33 surveys and 385,810 participants. PLOS ONE 16(5): e0250824. <https://doi.org/10.1371/journal.pone.0250824>

The suggested references have been added to the revised manuscript to more thoroughly recognise the previous work that has been undertaken in this area in Australia.

\## Methods

\### Data on smoking

**C3.6)** Relapse is common after only 1 month of cessation, see:

Herd, N., Borland, R., & Hyland, A. (2009). Predictors of smoking relapse by duration of abstinence: findings from the International Tobacco Control (ITC) Four Country Survey. Addiction, 104(12), 2088-2099.

therefore there is likely a lot of misclassification amongst those who had quit 1 or 2 years before the most recent measurement. If possible, the observation of smoking status could be censored to 2 years prior to the most recent survey in either the main analysis, or as a sensitivity analysis.

Your suggestion is in line with the analysis that we did in our previous paper on a European population (Pesce et al. PONE 2019). Such analysis was not implemented in the present manuscript due to the previous experience that the temporal trends were only marginally affected by this, probably due to a stable proportion of sustained quitters within the total of quitters over time (lines 240-244).

In the revised manuscript, we have added a new sensitivity analysis based on the recommended definition of “sustained quitting”. This has been described in the Methods section:

*As the risk of relapse is high in the first year after quitting, while it drastically drops afterwards (Garcia-Rodrigues et al.,* *Drug Alcohol Depend. 2013), we performed a sensitivity analysis defining smoking cessation as having quit smoking for at least two years (with person-years re-calculated accordingly), in order to exclude subjects who might have quit smoking just for a brief time before the survey.*

In the revised manuscript, we have reported on crude and adjusted rates of sustained smoking cessation (lines 338-340), which are included in **S1 Appendix** and reported below. While it is possible to appreciate a change in the rates of smoking cessation in both males and females, the study conclusions on trends were not affected by this (**Table B** and **Figure A**).

**Table B:** Crude rates of sustained smoking cessation per 1000/year (and person-years at risk), by sex, cohort and period. Cells with less than 100 person-years at risk were omitted.

|  | **Males aged**  **Age 36-50** | | | **Females**  **Age 36-50** | | |
| --- | --- | --- | --- | --- | --- | --- |
|  | BHS | TAHS | Pooled | BHS | TAHS | Pooled |
| 1930-1939 | 5.64  (1,241) |  | 5.64  (1,241) | 4.29  (233) |  | 4.29  (233) |
| 1940-1949 | 9.77  (2,662) |  | 9.77  (2,662) | 10.11  (791) |  | 10.11  (791) |
| 1950-1959 | 21.39  (4,021) |  | 21.39  (4,021) | 10.65  (1,972) |  | 10.65  (1,972) |
| 1960-1969 | 26.90  (4,237) |  | 26.90  (4,237) | 12.98  (2,774) |  | 12.98  (2,774) |
| 1970-1979 | 37.56  (2,316) |  | 37.56  (2,316) | 21.42  (1,727) |  | 21.42  (1,727) |
| 1980-1989 | 61.72  (1,118) | 40.68  (885) | 52.42  (2,003) | 37.64  (797) | 35.16  (768) | 36.42  (1,565) |
| 1990-1999 | 44.43  (3,061) | 28.36  (12,448) | 31.53  (15,509) | 44.11  (2,970) | 23.50  (10,851) | 27.93  (13,821) |
| 2000-2005 | 49.65  (1,732) | 34.67  (12,430) | 36.51  (14,162) | 54.69  (1,810) | 32.31  (11,267) | 35.41 (13,077) |

**Figure A.** Estimated trends in sustained smoking cessation with 95% confidence intervals, by sex.*

* blue lines: males; red lines: females. Generalized linear models with negative binomial distribution, logarithmic link function, an offset for log person-years, and adjusted for study group, age, and age^2^; time was modelled using natural splines with 3 equally spaced inner knots. The bump in the curves in 1975-1984 was due to having no person-years at risk for 1980 and 1981, and few cases of sustained quitting in general during the period

\### Statistical analysis

**C3.7)** The number of knots considered and the BIC for each model should be reported.

Thank you for bringing this to our attention. Your request has helped us identify an error in the selection of the number of knots in the analysis. We sincerely apologize.

In the original version of the manuscript, we applied 3 knots for all the analyses. However, for smoking initiation, the correct number of knots based on the minimum BIC criterion was 4 for males aged 11-15, 5 for females aged 11-15, and 4 for males aged 21-35. In the revised manuscript, we have reported the number of knots tested and the BIC value for each model in new **S2 Table** (also reported below).

While for males aged 21-35, there was no material difference in the results (new **Figure 3** below), the results changed for the 11-15 years old age groups, where a peak replaced a steady increase in the 1970s in both males and females. The revised analysis still highlights a very high rate of smoking initiation in the 70’s among young adolescents but it was reassuring to see evidence for a starting decline in initiation. We have revised the text of the manuscript accordingly.

**S2 Table.** Values of the Bayesian Information Criterion (BIC) for the different number of knots tested in the natural spline functions for period, by analysis and sex.

| **Analysis** | **Age group** | **N. of knots** | **BIC values for males** | **BIC values for females** |
| --- | --- | --- | --- | --- |
| Smoking initiation | 11-15 | 3 | 1771.26 | 1074.86 |
|  |  | 4 | 1766.38* | 1019.79 |
|  |  | 5 | 1771.91 | 1008.09* |
|  | 16-20 | 3 | 2299.95* | 2136.18* |
|  |  | 4 | 2300.24 | 2140.84 |
|  |  | 5 | 2305.79 | 2146.94 |
|  | 21-35 | 3 | 1345.23 | 1549.24* |
|  |  | 4 | 1344.38* | 1553.75 |
|  |  | 5 | 1347.04 | 1560.51 |
| Smoking cessation  (main analysis) | 36-50 | 3 | 2922.06* | 2205.60* |
|  |  | 4 | 2924.55 | 2209.95 |
|  |  | 5 | 2932.40 | 2215.96 |
| Smoking cessation  (sensitivity analysis) | 36-50 | 3 | 2824.25* | 2148.05* |
|  |  | 4 | 2825.84 | 2151.11 |
|  |  | 5 | 2833.71 | 2157.48 |

* selected number of knots from the model with the lowest BIC value

**Fig 3.** Estimated trends in smoking initiation with 95% confidence intervals for males (panel A) and females (panel B), by age group*

* green lines: age 11–15; orange lines: age 16–20; grey lines: age 21–35. Generalized linear models with negative binomial distribution, an offset for log person-years, and adjusted for study group, age, and age^2^; time was modelled using natural splines with equally spaced inner knots (see **S2 Table** for the number of knots)

**C3.8)** The predictions of the model should also be compared to the data used; e.g. for each data point, a _prediction_ interval (not confidence interval) should be provided, then outliers and the coverage of the intervals should be assessed.

We have conducted a descriptive analysis on temporal trends in the rates of smoking initiation and smoking cessation using natural splines to model time. The analysis was aimed at describing and visualizing the observed temporal patterns in the study area. For such purposes, we believe it was correct to report confidence intervals, which quantify the uncertainty around the estimated rates based on sample data, under the assumption that the underlying model was correct. Since we were not making out-of-sample predictions or forecasting future observations, we believe we do not need to estimate a prediction interval, which would be used to quantify the uncertainty around a future observation or out-of-sample prediction by accounting for both the uncertainty in the parameter estimate and the inherent variability of future observations. Forecasting was beyond the scope of our analysis, considering the study design and sparse data issues for more recent periods.

\## Discussion

**C3.9)** 1st sentence: Revise, the data suggests that 57% of people regularly smoked over the entire period, but at any one point in the period it may have been lower or higher than this.

2nd sentence: Revise: "Between 1930 and 2005 only 27% of those who smoked in the age range 35-50 years stopped smoking"

We agreed with these comments, now implemented.

\### Smoking initiation

**C3.10)** 1st paragraph: Reference [13] in the manuscript did not use any local sources of data on age at initiation (see its supplementary material); it would be better to use the published sources of information which include the National Drug Strategy Household Survey (NDSHS) 2019 (reference [3] in manuscript) or the reference to Vaneckova et al, 2019, I provided earlier also provides local data on the proportion of those who (ever) smoked that start smoking by a given age. The NDSHS 2019 also indicates that fewer than 20% of 18-24 year olds smoke (10% daily, and 7% occasional), therefore the final sentence should be amended.

The references suggested have now been added to the Discussion, at the end of Paragraph 1 of the Smoking Initiation Section (lines 362-368):

*The Australian 2022-23 National Drug Strategy Household Survey [23] showed that, for those aged 18-24 years, 10.5% of males and 8.3% of females currently smoke, continuing a downward trend from 2001 (34.6% and 29.6% respectively). These data agree with our data on downward and converging trends for age at initiation of smoking, so that in the most recent birth cohort (1980-89) mean age at smoking initiation was 18.0 years for men and 17.4 for women [24].*

\## Smoking cessation

**C3.11)** Line 316: remove repeated 'effective'.

Line 321: Reference [9] does not estimate quit rates; therefore the comparison is not valid.

Amended.

**C3.12)** A comparison here could be made to the results provided in the pre-print above (Wade et al, 2022). Perhaps, more importantly, to other studies such as Holford et al, 2014, or Gartner et al, 2009.

Thank you for suggesting these important studies which have been included in the revised manuscript (lines 392-404).

*Using modelling methods and large data bases to calculate current and future prevalence and cessation rates of smoking, Holford et al. showed that cessation rates were higher in later birth cohorts and increased with age in all cohorts in both males and females in the US [13]. However, using similar methods based on Australian data, Gartner et al. found a decrease in cessation rates in younger (20-30 years) males and females in the period 2001-2007 [14]. Trends were variable in age group 31-50 years during the same period, with an increase in cessation rates in males and a slight decrease in females. In our study, fully adjusted cessation rates for different age groups could not be calculated due to smaller sample sizes. However, the crude rates of cessation showed a decrease in younger (16-35 years) males during 2000-2005 and in older (51-65 years) males and females during 1990-1999, and a continuing upward trend in younger females (16-50 years) (S7 and S8 Tables).*

**C3.13)** A critical missing piece of context is that the National Tobacco Campaign (1997-2001) was in full effect during the period where it has been estimated in this manuscript that cessation rates were in decline. This requires some explanation; either the authors would need to substantiate why cessation rates were in decline or explain why their model disagrees with a fairly reasonable expectation that they would (likely) be increasing.

One potential explanation I can offer is the reliance on adjustment for age (and age^2) in a period where less data on cessation from younger smokers is available (i.e. fewer eventual relapsers are reporting that they had quit). As mentioned above, a sensitivity analysis which censors observations of cessation events 2 years before the surveys might be helpful here.

We acknowledge that this is an important point of discussion for the present manuscript.

The new analyses provided in the revised manuscript support the stability of the study findings on cessation rates in the age range 36-50 years, where results for sustained cessation were fairly consistent with the main analysis (**S1 Appendix**).

One of the reasons for stable/decreasing rates of smoking cessation in our study that did not apparently match with observations by others could be differences in study design and outcome measures assessed. Results from repeated population surveys assessing prevalence rates of former smoking could diverge from results from our study, based on pooling of fixed cohort studies assessing cessation rates. Stable/decreasing rates of smoking cessation over time in our study could be related to an increasing proportion of hard-core smokers over time. Stable/decreasing cessation rates over time indicate a slow-down of cessation behaviours in the study cohort, which would still be compatible with an increasing quit proportion in the population, i.e. an increasing cumulative count of former smokers over a relatively stable count of ever smokers.

Other potential explanations include the restricted age range in our study (although unlikely, since the crude rates for the other age groups showed similar behaviour), uncertainty of trends in the most recent periods due to less data for the latest years, as well as the heterogeneity between our study samples and the broader Australian population. These explanations have been reported in the revised Discussion (lines 412-421).

\### Strengths and limitations

**C3.14)** The age-stratified analysis of initiation doesn't appear to account for the differences in distribution of age in later periods (e.g. for the period 1990-1999, no survey contained an individual aged 21 in that period according to Table 2); thus the analysis would under-estimate the initiation rate.

Another (likely) source of bias in the initiation rate is survivor bias. This would lead to under-estimation of the initiation rate in older cohorts.

Misclassification of those who ever smoked (versus never smoked) would also lead to under/mis-estimation of initiation rates in earlier cohorts; this misclassification has been observed in the Busselton study itself:

- Visalpattanasin, P., Wearne, K. L., & Armstrong, B. K. (1987). Trends in the uptake of smoking in Busselton, Western Australia. Australian and New Zealand Journal of Public Health, 11, 21-s.

Wade et al, 2022, shows (Table S2-10,S2-12) that not accounting for this effect can materially impact the estimate of both the proportion that initiate within a cohort and the cessation rate in a calendar year.

Thanks for this comment. We agree that the analysis could be prone to sources of bias that we were unable to account for due to the types of data available. In the revised version of the manuscript, we have acknowledged that a study limitation arises from the secondary use of data that were not expressly collected for the purpose of investigating temporal trends in smoking habits. This could result in bias due to imbalance of participants across ages and birth cohorts (see lines 439-444).

**C3.15)** An obvious, but not stated, limitation is that the cohorts are not random samples from the complete Australian population; they are more representative of 'regional' Australia than metropolitan Australia and given that regional status is an influence on smoking behaviour - this suggests some care should be taken when generalising to the Australian population.

We have included a comment about ‘regionality’ in the revised manuscript, Strengths and Limitations (lines 460-462):

*Regional centres have 3-5% higher prevalence of current smoking compared with major cities.[29]*

\### Implications

**C3.16)** Line 382: The 'hard core' smokers hypothesis is not considered infallible, see

- Skinner, A., Occhipinti, JA. & Osgood, N.D. A dynamic modelling analysis of the impact of tobacco control programs on population-level nicotine dependence. Sci Rep 11, 1866 (2021).

https://doi.org/10.1038/s41598-021-81460-9

We agree. The term “hard core” has been replaced.

**C3.17)** Line 385-387: At face value I agree that initiation and cessation rates are less favourable in Aboriginal and Torres Strait Islander communities, this was not the focus of reference [23] nor is it covered in [24] for mental illness.

Enduring high prevalence in older populations, described in Lovett et al, 2017, are supportive of the need for more focus on cessation in Aboriginal and Torres Strait Islander people. I would rework this last sentence using the following text from the Conclusions of [23] as a starting point, with some simplifications needed to put it in the context of the study in the manuscript:

The challenges for indigenous people are much greater and include poverty, marginalization, challenges in accessing resources, high rates of smoking, and acceptance of smoking in families and communities. This review has underscored the complexity of achieving smoking cessation and the need to collaboratively develop interventions that are acceptable and appropriate to local populations.

We have added a final sentence in Strength and Limitations to address this point (lines 512-517):

*Multiple factors such as high levels of exposure to smoking and marginalization contribute to the challenge of reducing smoking prevalence in Indigenous communities. The development of acceptable and culturally appropriate interventions is required to address these complex, overlapping issues.*

**C3.18)** Line 393: A more up-to-date reference on the use of vaping amongst youth in Australia is provided by the ASSAD survey I mentioned above, e.g. 15.7% of students reported using vapes in the past month in 2022, up from 4.2% in 2017.

The sentence has been adjusted to reflect the more recent data (lines 521-522):

*e-Cigarette use in the past month has increased from 4.2% in 2017 to 15.7% in 2022, particularly in young adults.[3]”*

\## Conclusions

**C3.19)** Line 411: I'd leave out 'political conflict' unless further commentary on the difficulty in adoption of the FCTC, re-commitment to MPOWER, and recent changes in vaping policy are provided in the Discussion.

The phrase “political conflict” has been removed.

**C3.20)** I'd also pivot away from saying 'unacceptably high level of tobacco use' when this is being written by and from the perspective of people who do not belong to the groups being talked about (it could read as an admonishment rather than coming from a place of empathy). An alternative might be "The persistence of (health) inequity caused by smoking amongst marginalised people suggests..." although I recognise it isn't the plainest English.

Agree. This sentence has been altered as suggested (lines 537-542):

*The persistence of an health inequity due to smoking, particularly among susceptible groups, suggests the need for identifying more specific risk factors for smoking initiation,[19] developing better targeted approaches for smoking cessation[27] and addressing the emerging risks associated with e-cigarettes and increased mental health disorders.*

Lastly, I would amend some of the language used throughout, replacing 'smoker' with 'person who smoked' or similar, as per guidance I have received about labelling and stigmatisation, also see:

- Hefler M, Durkin SJ, Cohen JE, et al. New policy of people-first language to replace ‘smoker’, ‘vaper’ ‘tobacco user’ and other behaviour-based labels. Tobacco Control 2023; 32:133-134.

- Renee D Goodwin, Lisa K Walker, Time to Stop Using the Word “Smoker”: Reflecting on the Role of Language in Advancing the Field of Nicotine and Tobacco Research, Nicotine & Tobacco Research, Volume 24, Issue 12, December 2022, Pages 1847–1848. https://doi.org/10.1093/ntr/ntac218

Thanks, an important comment. We have revised the terminology.
