# Supplementary material for: Trends in smoking initiation and cessation over a century in two Australian cohorts
Source: PLoS One. 2024 Sep 19;19(9):e0307386. doi: 10.1371/journal.pone.0307386 (PMC11412490; doi:10.1371/journal.pone.0307386)
Supplement: Attachment — Submitted filename: responses to comments (R2).docx [file pone.0307386.s014.docx]

**PONE-D-24-00230**

**Trends in smoking initiation and cessation over a century in two Australian cohorts**

Responses to comments

**Journal Requirements**

Please review your reference list to ensure that it is complete and correct. If you have cited papers that have been retracted, please include the rationale for doing so in the manuscript text, or remove these references and replace them with relevant current references. Any changes to the reference list should be mentioned in the rebuttal letter that accompanies your revised manuscript. If you need to cite a retracted article, indicate the article’s retracted status in the References list and also include a citation and full reference for the retraction notice.

We are not aware of any retracted article in the reference list. In the revised reference list, we have amended some inconsistencies as recommended by Reviewer #3. We believe that the reference list is now complete and correct.

**Reviewer #1**

Thank you for providing further feedback on the manuscript. We have revised it accordingly. In particular, we have downplayed the study conclusions in the abstract and manuscript as suggested.

C1.1) Line 92: The Surgeon General Report was published in 1964.

Apologies for this mistake, we have now amended it.

C1.2) I still worry about drawing strong conclusions about prevention programs today based on such old data.
Line 543-544: Drop this statement. It isn’t really based on your data and doesn’t add anything.

We acknowledged your perplexity and accepted your suggestion.

C1.3) I still don’t understand how the applicants actually did the pooling. It sounds like they just dumped all the data into a single file. Please be more precise.

Also, did the surveys account for complex survey design? If so, how was that considered in the analysis and pooling?

You are right that the description could be further improved.

The individual data of unique participants were used to generate datasets including the count of individuals starting or quitting smoking and the total number of participants “at risk”, for each combination of sex, age (rounded to the nearest integer), and period (calendar year). This was first done for each cohort, and then the two resulting datasets were merged.

The data from each cohort remained distinct in the final “merged” dataset, in order to enable adjustment by cohort in the pooled analysis. Readers can access **S1 Dataset** and **S2 Dataset** in the supplementary materials to get a full understanding of how the pooled datasets look like.

We report below an extract of **S1 Dataset** on smoking initiation, after transforming the CSV format into a data matrix for the sake of clarity. Empty cells for the “D” variable indicate 0 new smokers for that cohort, sex, age, and period among “pop” people at risk.

| **cohort** | **sex** | **A** | **P** | **D** | **pop** |
| --- | --- | --- | --- | --- | --- |
| BHS | Male | 22 | 1951 |  | 20 |
| TAHS sib | Male | 22 | 1990 |  | 9 |
| TAHS sib | Male | 14 | 1970 | 26 | 343 |
| TAHS sib | Male | 32 | 1993 |  | 2 |
| BHS | Male | 15 | 1921 | 8 | 57 |
| BHS | Male | 16 | 1922 | 5 | 49 |
| TAHS sib | Male | 26 | 1970 |  | 7 |
| BHS | Male | 20 | 1948 | 7 | 27 |
| TAHS sib | Male | 20 | 1964 |  | 8 |
| TAHS sib | Male | 32 | 1999 | 1 | 81 |
| BHS | Male | 17 | 1909 |  | 9 |
| TAHS sib | Male | 17 | 1980 | 40 | 361 |
| BHS | Male | 29 | 1935 |  | 17 |
| BHS | Male | 34 | 1970 |  | 22 |
| BHS | Male | 35 | 1941 |  | 17 |
| TAHS sib | Male | 34 | 1980 |  | 21 |
| BHS | Male | 30 | 1988 |  | 44 |
| BHS | Male | 32 | 1929 |  | 2 |
| TAHS prob | Male | 28 | 1988 |  | 1 |
| BHS | Male | 33 | 1995 |  | 55 |

We have now rewritten the description of data pooling as follows:

*Pooling involved generating aggregated datasets comprising the numbers of cases of smoking initiation/cessation and person-years at risk, categorised by cohort, gender, age of participants (rounded to the nearest integer), and period (calendar year). The pooled datasets are available as* ***S1 and S2 Datasets****.*

TAHS data represent the follow-up of a fully enumerated cohort, which is equivalent to a 100% sampling. BHS was a repeated cross-sectional survey, drawing simple random samples of the target population. We have not used complex survey design since we assumed that pooling data from studies based on simple random sampling would be less prone to bias, compared to pooling data that applied complex sampling schemes. However, we have performed separate analyses by cohort, and the results for TAHS and BHS were consistent. Similar findings from the two cohorts are reassuring that differences in study designs are unlikely to have severely biased our investigation. Issues regarding imbalance of participants across ages and birth cohorts due to attrition of the cohorts over time are discussed in the Discussion.

**Reviewer #3**

R3.1) The authors have comprehensively and accurately responded to all my feedback, with only one minor exception that I'll put on the record to help the authors in any further investigation. Otherwise, the manuscript is worthy of publication and the discussion provides interesting comparison of trends in starting and stopping smoking between settings (US, Europe, Australia).

\# Minor feedback

Original feedback: C3.8) The predictions of the model should also be compared to the data used; e.g. for each data point, a _prediction_ interval (not confidence interval) should be provided, then outliers and the coverage of the intervals should be assessed.

Author's response: We have conducted a descriptive analysis on temporal trends in the rates of smoking initiation and smoking cessation using natural splines to model time. The analysis was aimed at describing and visualizing the observed temporal patterns in the study area. For such purposes, we believe it was correct to report confidence intervals, which quantify the uncertainty around the estimated rates based on sample data, under the assumption that the underlying model was correct. Since we were not making out-of-sample predictions or forecasting future observations, we believe we do not need to estimate a prediction interval, which would be used to quantify the uncertainty around a future observation or out-of-sample prediction by accounting for both the uncertainty in the parameter estimate and the inherent variability of future observations. Forecasting was beyond the scope of our analysis, considering the study design and sparse data issues for more recent periods.

Reply: I would still recommend (perhaps in future investigations) that within-sample predictive checks are done on models. This is not to assess how the model performs out of sample. For example, if less (or more) than 95% of the (within sample) prediction intervals contain the observed value, then it is plausible/likely that the model is missing detail or (in very rare cases) was not fit correctly - this also has bearing on whether the confidence intervals themselves have the stated coverage.

Even though your analysis is not Bayesian, a relevant resource is Bayesian Data Analysis by Gelman:

Gelman, A., Carlin, J. B., Stern, H. S., Dunson, D. B., Vehtari, A., & Rubin, D. B. (2014). Bayesian Data Analysis.

Thank you for providing this clarification. We are grateful for the time you dedicated in thoroughly reviewing the manuscript and for the present reference.

R3.2) # Data availability
I'd recommend confirming that the cell-sizes in the spreadsheet provided do not violate any data confidentiality or other Ethics agreements about using the data. Line data does not need to be made available according to my own understanding of the PLOS ONE data policy.

Our datasets include the counts of individuals starting (**S1 Dataset**) or quitting (**S2 Dataset**) smoking and the total number of participants “at risk” for each specific stratum. The strata result from combining cohort (TAHS probands/siblings and BHS), sex, age (rounded to the nearest integer), and period (calendar year). The BHS cohort began with a simple random sample of the population living in Busselton, whereas the TAHS cohort encompassed the entire eligible population originally residing in Tasmania. The sampling covered such broad geographical areas that we deem re-identification virtually impossible. Therefore, we do not foresee any confidentiality issues.

R3.3) # Some fixes for references

1. I spotted some inconsistency with shortened journal name vs full name (and capitalisation of PLoS ONE vs PLOS ONE)
2. Check capitalisation in reference 26, and title case (?) in 28 ,34, 41, and 43.
3. pdf links seemed not to work (probably just a rendering issue).

We have now standardised the capitalisation of article titles to match that used in PubMed, harmonised abbreviated journal names according to the [National Library of Medicine Catalog](https://www.ncbi.nlm.nih.gov/nlmcatalog/journals), and corrected a few non-functional URL links.
